# Proximal deep vein thrombosis and pulmonary embolism in COVID-19 patients: a systematic review and meta-analysis

**DOI:** 10.1186/s12959-021-00266-x

**Published:** 2021-03-09

**Authors:** Gregoire Longchamp, Sara Manzocchi-Besson, Alban Longchamp, Marc Righini, Helia Robert-Ebadi, Marc Blondon

**Affiliations:** 1 Department of Visceral Surgery, Faculty of Medicine and Geneva University Hospitals, Geneva, Switzerland; 2 Division of Angiology and Haemostasis, Geneva University Hospitals and Faculty of Medicine, Rue Gabrielle-Perret-Gentil 4, 1205 Geneva, Switzerland; 3 Department of Vascular Surgery, Centre Hospitalier du Valais Romand de l’Hôpital du Valais (site de Sion), Sion, Switzerland; 4 Department of Vascular Surgery, Centre Hospitalier Universitaire Vaudois and University of Lausanne, Lausanne, Switzerland; 5 Department of Biomedical Sciences, University of Lausanne, Lausanne, Switzerland

**Keywords:** Coronavirus, Venous thromboembolism, Thrombosis pulmonary embolism

## Abstract

**Background:**

COVID-19 appears to be associated with a high risk of venous thromboembolism (VTE). We aimed to systematically review and meta-analyze the risk of clinically relevant VTE in patients hospitalized for COVID-19.

**Methods:**

This meta-analysis included original articles in English published from January 1st, 2020 to June 15th, 2020 in Pubmed/MEDLINE, Embase, Web of science, and Cochrane. Outcomes were major VTE, defined as any objectively diagnosed pulmonary embolism (PE) and/or proximal deep vein thrombosis (DVT). Primary analysis estimated the risk of VTE, stratified by acutely and critically ill inpatients. Secondary analyses explored the separate risk of proximal DVT and of PE; the risk of major VTE stratified by screening and by type of anticoagulation.

**Results:**

In 33 studies (*n* = 4009 inpatients) with heterogeneous thrombotic risk factors, VTE incidence was 9% (95%CI 5–13%, *I*^*2*^ = 92.5) overall, and 21% (95%CI 14–28%, *I*^*2*^ = 87.6%) for patients hospitalized in the ICU. Proximal lower limb DVT incidence was 3% (95%CI 1–5%, *I*^*2*^ = 87.0%) and 8% (95%CI 3–14%, *I*^*2*^ = 87.6%), respectively. PE incidence was 8% (95%CI 4–13%, *I*^*2*^ = 92.1%) and 17% (95%CI 11–25%, *I*^*2*^ = 89.3%), respectively. Screening and absence of anticoagulation were associated with a higher VTE incidence. When restricting to medically ill inpatients, the VTE incidence was 2% (95%CI 0–6%).

**Conclusions:**

The risk of major VTE among COVID-19 inpatients is high but varies greatly with severity of the disease. These findings reinforce the need for the use of thromboprophylaxis in all COVID-19 inpatients and for clinical trials testing different thromboprophylaxis regimens in subgroups of COVID-19 inpatients.

**Trial registration:**

The review protocol was registered in PROSPERO International Prospective Register of Systematic Reviews (CRD42020193369).

**Supplementary Information:**

The online version contains supplementary material available at 10.1186/s12959-021-00266-x.

## Background

Coronavirus disease 2019 (COVID-19) is caused by the severe acute respiratory syndrome coronavirus 2 (SARS-CoV2). COVID-19 is characterized by vascular inflammation, with evidence of viral elements within endothelial cells [1]. Initial reports suggested an abnormal activation of coagulation, in particular with highly elevated D-dimer levels [2], which predicted a poor clinical prognosis and death [3, 4].

Since the start of the outbreak, several studies have highlighted a high risk of venous thromboembolism (VTE) in inpatients with COVID-19, up to 35–85% and oftentimes despite pharmacological thromboprophylaxis [5, 6]. However, such large estimates may emanate from the inclusion of small thrombi of uncertain clinical relevance, such as distal deep vein thrombosis (DVT), and may be more prone to being published with high visibility and publicized largely. A similar situation may be found in the lungs, where the vascular filling defects reported on computed tomography pulmonary angiography (CTPA) might reflect “immuno-thrombosis” or local “pulmonary thrombosis” rather than “classic” pulmonary embolism (PE).

Therefore, we aimed to systematically review the published literature on the risk of objectively diagnosed PE and/or proximal DVT – representing major VTE – in hospitalized patients with COVID-19. This information should help clinical decision and guidance with regards to thromboprophylaxis.

## Methods

### Search strategy and study eligibility

We performed this systematic review and meta-analysis in concordance with the Preferred Reporting Items for Systematic Reviews and Meta-Analyses (PRISMA) guidelines (additional table 1) [7]. The review protocol was registered in PROSPERO International Prospective Register of Systematic Reviews (CRD42020193369).

We searched for original articles in English language, in which the risk of major VTE could be estimated among COVID-19 inpatients. As such, we excluded case reports or case series of VTE. Studies were assessed for eligibility, regardless of the sample size, population group/subgroup of COVID-19 inpatients, and study design (observational or interventional, retrospective or prospective). Studies were included if SARS-CoV2 was confirmed with a PCR or based on clinical and radiological examinations [8].

Only peer-reviewed literature was appraised, published from January 1st, 2020 to June 15th, 2020, in Pubmed/MEDLINE, Embase, Web of science, and Cochrane. The electronic search was supplemented by a search of the Lancet COVID-19 Ressource Centre, JAMA COVID-19, the New England Journal of Medicine Coronavirus, the use of the “similar articles” function in Pubmed, and the screening of the references list of all relevant articles (additional file 1).

### Outcomes

The primary outcome was the risk of major VTE, defined as any objectively confirmed PE and/or proximal DVT. Proximal DVT was defined as lower limb DVT occurring in the popliteal, femoral, iliac veins and/or inferior vena cava, regardless of the presence of a catheter, and confirmed by compression ultrasound (CUS) or CT phlebography. We therefore excluded distal DVT (below the popliteal trifurcation) and upper extremity DVT, the latter being almost always catheter-related and not associated with the same embolic potential as lower limb proximal DVT. PE had to be objectively confirmed by CTPA, ventilation/perfusion scintigraphy, the presence of a proximal DVT on lower limb CUS in a patient with clinical suspicion of PE, or a high clinical suspicion of PE with an acute right ventricular dilatation on echocardiography [9].

Secondary analyses explored the separate risk of proximal DVT and of PE, as well as the risk of major VTE stratified by the presence or absence of VTE screening tests and by anticoagulation regimen.

### Data extraction and quality assessment

The search, data extraction, and quality assessment were independently conducted in pairs by four authors (GL, AL, SMB and MB). Any disagreements were discussed until a consensus was reached, and solved by a third author (MR) whenever necessary.

Data were entered into a predesigned database, and included information on study design, setting, inclusion/exclusion criteria, patients characteristics, diagnostic methods for COVID-19, VTE diagnosis, VTE screening strategy, and the use of therapeutic anticoagulation or thromboprophylaxis. Whenever necessary, an email was sent to the corresponding author for additional details (of the 24 authors contacted, 15 answered).

The risk of bias was assessed using the Newcastle-Ottawa scale for non-randomized clinical trials (additional table 2) [10].

### Statistical analysis

The primary analysis estimated the risk of major VTE among studies including all inpatients – general medical wards and critical care units – and among studies including only intensive care unit (ICU) patients. This was computed by dividing the number of major VTE by the number of patients at risk at the start of the study. We did not consider Kaplan-Meier estimates, given their overestimating bias in case of competing risk of death.

In the secondary analyses, we explored the separate risk of proximal DVT and of PE; the heterogeneity; the risk of major VTE with and without screening for DVT; the risks of VTE among patients receiving therapeutic anticoagulation, thromboprophylaxis and no anticoagulant treatment; and the risk of VTE among medical patients, by excluding ICU patients.

In a sensitivity analysis, we repeated the primary analysis in a restricted sample of high-quality studies defined as retrospective or prospective studies with an unselected group of medical and/or ICU patients, with a reported ≥80% of PCR positivity and a reported mean follow-up of at least 7 days.

All meta-analytic estimates were pooled using random effect models, because of expected substantial heterogeneity due to different sampling characteristics. Risks (proportions) observed in studies were transformed with the Freeman-Tukey double arcsin method to remove the dependence of the variance on the mean of the transformed proportion and to correct for overdispersion of estimates [11]. Heterogeneity itself was measured by using the *I*^*2*^, describing the proportion of the observed between-studies variation not due to chance. Small-study effects were explored graphically with funnel plots and with Egger’s tests.

All analyses were conducted in Microsoft Excel, Stata version 11 (including with the package *metaprop)* and R.

## Results

### Study selection and characteristics

**Fig. 1:**
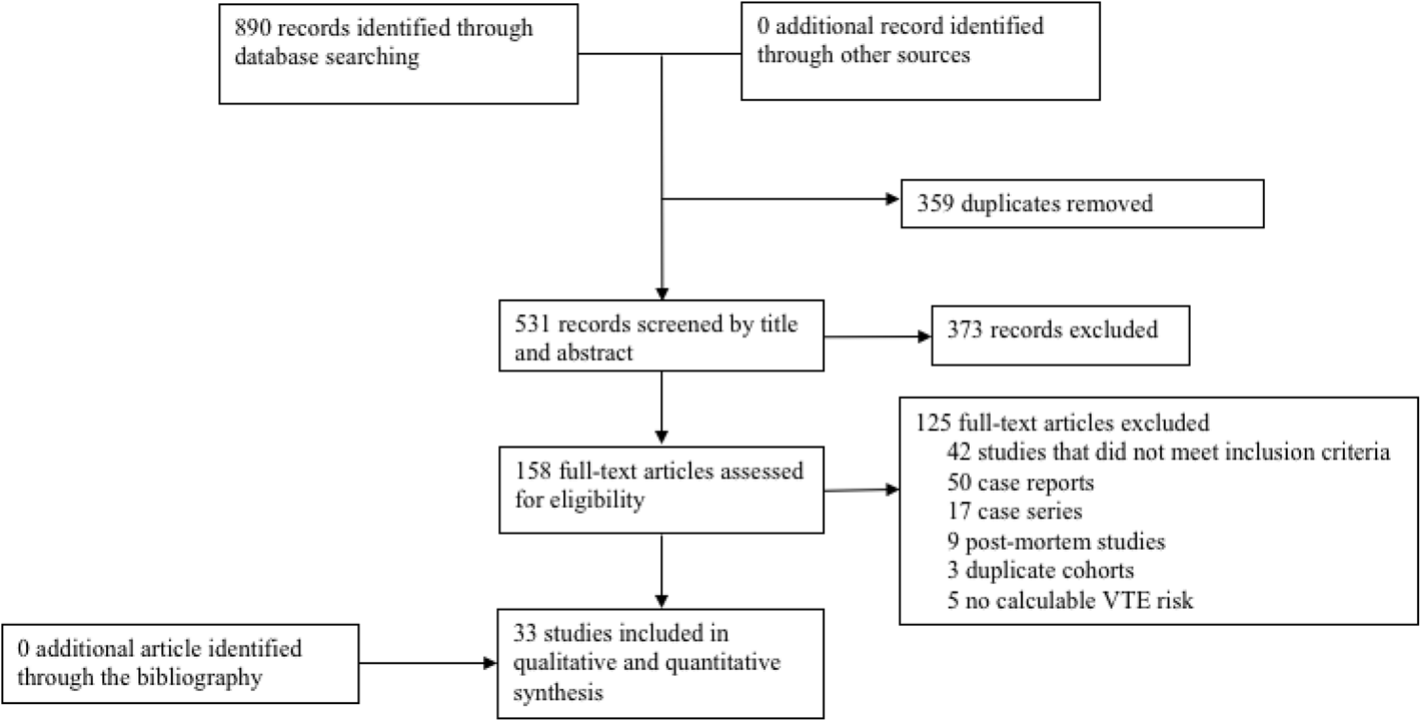
Preferred Reporting Items for Systematic Reviews and Meta-analyses (PRISMA) flowchart showing selection of publication for review and meta-analysis. VTE = venous thromboembolism

Electronic database searches identified 890 titles and abstracts (Fig. 1). After removal of duplicates, 531 papers remained. Reviewing titles and abstracts resulted in 158 potential articles to be included. After full-text reading, 33 studies [6, 12–43] were included in the systematic review and meta-analysis (substantial agreement: Cohen’s Kappa = 0.79). No additional articles were identified by other sources of screening or by reviewing the relevant reference lists.

28 studies [13–27, 29–32, 34–41, 43] were conducted in Europe, three [12, 28, 33] in the USA, and two [6, 42] in Asia (Table 1). 19 [12–15, 17, 21–23, 26, 28–31, 33–35, 37, 38, 40] were retrospective cohorts, 12 [16, 18, 20, 24, 25, 27, 32, 36, 39, 41–43] were prospective cohorts, and two [6, 19] were cross-sectional studies. 19 [6, 13, 15, 16, 21, 23, 27–30, 32, 33, 36–41, 43] reported VTE in the ICU, while 14 studies [12, 14, 17–20, 22, 24–26, 31, 34, 35, 42] reported on a mixed population of medical ward and ICU patients.

**Table 1:** Characteristics of included studies

	All studies, mean (range) or n (%) (***n*** = 33)	Mixed ward + ICU studies, mean (range) or n (%) (***n*** = 14)	ICU studies, mean (range) or n (%) (***n*** = 19)
Continent			
- Europe	28 (84.9%)	12 (85.7%)	16 (84.2%)
- USA	3 (9.1%)	1 (7.1%)	2 (10.53%)
- Asia	2 (6.1%)	1 (7.1%)	1 (5.26%)
Study design			
- Retrospective cohort	19 (57.6%)	8 (57.1%)	11 (57.9%)
- Prospective cohort	12 (36.4%)	5 (35.7%)	7 (36.84%)
- Cross-sectional	2 (6.1%)	1 (7.1%)	1 (5.3%)
NOS			
-	2 (6.1%)	0	2 (10.5%)
-	8 (24.2%)	4 (28.6%)	4 (21.1%)
-	12 (36.4%)	6 (42.9%)	6 (31.6%)
-	7 (21.2%)	2 (14.3%)	5 (26.3%)
-	4 (12.1%)	2 (14.3%)	2 (10.5%)
n total	4009	2747	1262
n per study	121 (10–785)	196 (25–785)	66 (10–184)
n VTE total	429	170	259
Age, years	64 (57–69) [missing = 10]	66 (61–71) [missing = 4]	63 (57–70) [missing = 6]
Women	31.8% (13.8–48.3%) [missing = 8]	34.2% (13.8–48.3%) [missing = 3]	30.0% (16.7–45.8%) [missing = 5]
CVD	21.3% (8.8–52%) [missing = 19]	23.0% (11.9–52%) [missing = 9]	20.4% (8.8–48%) [missing = 10]
HTN	48.0% (32–84.6%) [missing = 17]	43.0% (32–62.5%) [missing = 7]	51.8% (38.2–84.6%) [missing = 10]
DM	26.8% (4–44.6%) [missing = 15]	19.2% (8–30.8%) [missing = 6]	32.9% (4–44.6%) [missing = 9]
Body-mass index, kg/m2	28.9 (23.6–34.8) [missing = 18]	27.0 (23.6–30) [missing = 8]	30.2 (27.5–34.8) [missing = 10]
Cancer	6.2% (0–20%) [missing = 17]	8.3% (3.5–20%) [missing = 6]	4.0% (0–8%) [missing = 11]
Personal history of VTE	3.0% (0–7.6%) [missing = 19]	2.9% (0–7.0%) [missing = 8]	3.0% (0–7.6%) [missing = 11]
Smoking	19.1% (3.1–43.8%) [missing =24]	15.6% (3.1–43.8%) [missing = 9]	2.5% (1.8–3.0%) [missing = 15]
Laboratory testing			
- Baseline D-dimer, μg/L	2352 (394–8300) [missing =14]	1808 (458–3700) [missing = 6]	2748 (394–8300) [missing = 8]
- Baseline fibrinogen, g/L	6.5 (4.1–9) [missing =17]	5.2 (4.8–5.9) [missing = 11]	6.8 (4.1–9) [missing = 8]
- Baseline platelets, × 10 [9]/L	233 (187–318) [missing = 18]	235 (187–286) [missing = 8]	232 (200–319) [missing = 10]
Anticoagulation			
- Chronic anticoagulation	7.4% (0–26.9%) [missing = 21]	6.8% (0–16%) [missing = 9]	7.8% (0–27.0%) [missing = 12]
- Therapeutic anticoagulation during the study	10.4% (0–69.2%) [missing =10]	4.3& (0–16%) [missing = 6]	13.6% (0–6.9%) [missing = 4]
- Prophylactic anticoagulation during the study	83.8% (0–100%) [missing = 11]	80.4% (20–100%) [missing = 5]	85.9% (30.8–100%) [missing = 4]
ICU			
- ICU hospitalization	76.4% (6.3–100%) [missing = 7]	26.6% (6.3–53.8%) [missing = 5]	100% (100–100%)
- Mechanical ventilation	67.5% (10–100%) [missing = 11]	25.2% (11.3–25.6%) [missing = 8]	83.3% (10–100%) [missing = 3]
- Use of vasopressors	64.2% (32.1–88.5%) [missing = 25]	- [missing = 14]	64.2% (32.1–88.5%) [missing = 11]
- Hemodialysis	22.9% (14.7–37.1%) [missing = 27]	- [missing = 14]	22.9% (14.7–37.1%) [missing = 13]
- ECMO	16.9% (0–100%) [missing = 24]	0% (0–0%) [missing = 13]	19.1% (0–100%) [missing = 11]

ICU = intensive care unit, NOS = Newcastle-Ottawa scale, VTE = venous thromboembolism, CVD = cardiovascular disease, HTN = hypertension DM = diabetes mellitus, ECMO = extracorporeal membrane oxygenation

The 33 studies included 4009 patients, with a range of mean age of 57–69 years, and proportion of women of 13.8–48.3%. The prevalence of VTE risk factors varied widely: a personal history of VTE was reported in 3.0% of patients (range 0–7.6%), D-dimer levels ranged from 394 μg/L to 8300 μg/L. Detailed characteristics of individual studies are found in the additional tables 3 and 4.

### Quality assessment

The Newcastle-Ottawa scale was used to assess risk of bias in each included study (additional table 2), excluding the items referring to the control group. Additionally, none of the studies demonstrated that the outcome of interest was not present at the start of the study. All studies met criteria for outcome assessment; consequently, the score ranged from one to 5 stars. 12 [12, 20, 22–25, 28, 30, 34, 36, 38, 41] and 8 [6, 15, 18, 19, 26, 39, 42, 43] studies were allocated three and two stars, respectively (Table 1). 7 [14, 21, 29, 31–33, 40] and 4 studies [17, 27, 35, 37] reached 4 and 5 stars, respectively; while the lowest score (one star) was obtained by two studies [13, 16].

### Venous thromboembolism incidence

#### Primary analysis

**Fig. 2:**
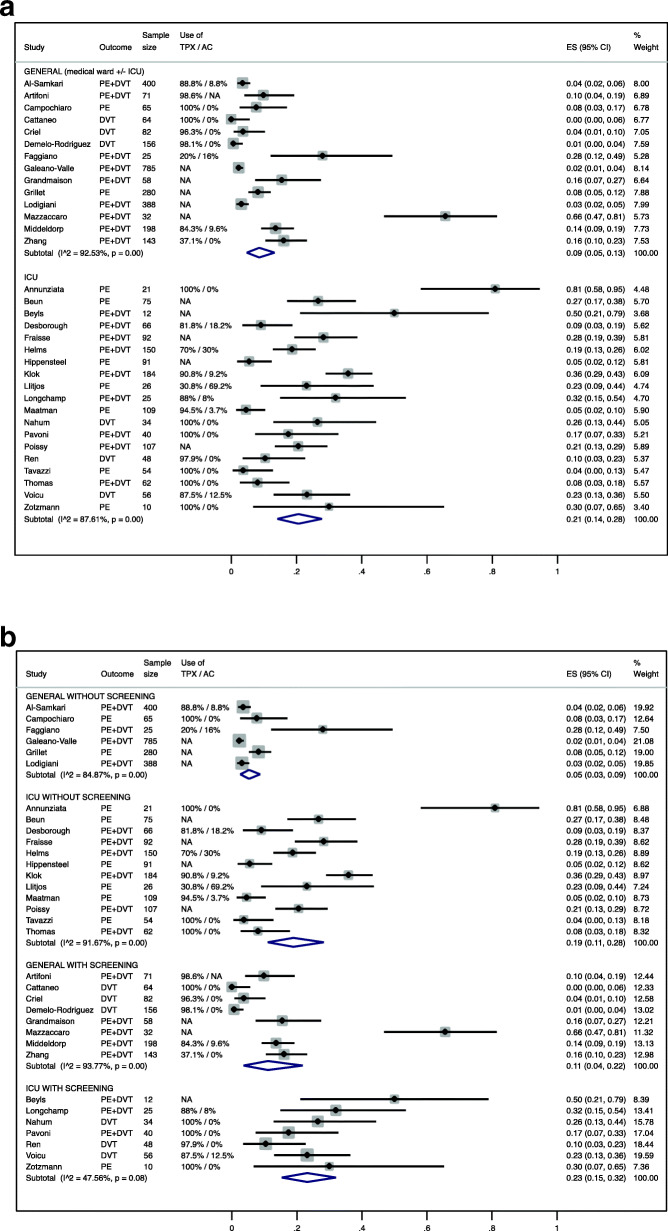
Forrest plot of the estimated incidence of VTE: 2a stratified by medical ward and ICU; 2b stratified by location and screening.TPX = thromboprophylaxis, AC = therapeutic anticoagulation, ICU = intensive care unit, PE = pulmonary embolism, DVT = deep vein thrombosis, NA = not available

In 14 studies [12, 14, 17–20, 22, 24–26, 31, 34, 35, 42] in COVID-19 patients from medical ward ± ICU, the pooled estimate of major VTE (proximal lower limb DVT and/or PE) incidence was 9% (95%CI 5–13%, *I*^*2*^ = 92.5) (Table 2, Fig. 2a). This rate was greater, at 21%, when restricting the analysis to patients hospitalized in the ICU (19 studies, 95%CI 14–28%, *I*^*2*^ = 87.6%, Table 2, Fig. 2a). The funnel plot appeared asymmetric for the 14 studies from medical ward ± ICU (Egger test *p* = 0.02), but appeared more symmetric for the 19 studies in the ICU (Egger test *p* = 0.47, additional Figs. 1a-b). This suggested that small studies tended to report higher risk estimates than larger studies, and were potentially more prone to be published.

**Table 2:** Meta-analytic estimates of the risk of VTE, stratified by location

	VTE		DVT		PE	
	ES (95%CI)	I^**2**^	ES (95%CI)	I^**2**^	ES (95%CI)	I^**2**^
**Medical ward ± ICU**	9% (5–13%)	92.5%	3% (1–5%)	87.0%	8% (4–13%)	92.1%
**ICU**	21% (14–28%)	87.6%	8% (3–14%)	87.6%	17% (11–25%)	89.3%

*VTE* venous thromboembolism, *DVT* deep venous thrombosis, *PE* pulmonary embolism, *ICU* intensive care unit

#### Secondary analyses

##### Proximal DVT

Proximal lower limb DVT confirmed by CUS was reported in 24 studies [6, 12, 14, 16, 18–25, 27, 29, 31, 32, 34–38, 40–42]; with a pooled estimated incidence of 3% (95%CI 1–5%) in the medical ward ± ICU patients, and 8% (95%CI 3–14%) when restricting to ICU patients (Table 2, additional figure 2a).

##### Pulmonary embolism

PE was reported in 27 studies, as confirmed by CTPA [12–17, 21–35, 37–40, 42, 43], or as a clinical suspicion of PE associated with a thrombus in the right atrium on echocardiography [16, 30, 31]. The estimated incidence of PE was also lower in the mix of medical and ICU patients (8, 95%CI 4–13%) than in the ICU only patients (17, 95%CI 11–25%) (Table 2, additional figure 3a). From the 330 PE reported in studies with data on location, 241 (73%) were segmental or more proximal (30 (9%) central, 39 (12%) lobar, 100 (30%) segmental, 72 (22%) unspecified) and 56 (17%) were subsegmental. The localization of PE was missing from 6 studies [24, 28, 30, 31, 33, 42], accounting for 33 PE (10%).

##### Heterogeneity

We explored the heterogeneity found in all analyses, which emanated from different inclusion criteria, definition of VTE, duration of follow-up, screening, use of thromboprophylaxis. When examining outliers, Annunziata et al. [13] reported an 81% risk of PE (95%CI 58–95%) in a small, highly selected sample of 21 ICU patients with disseminated intravascular coagulation. Beyls et al. [16], another outlier, reported a high DVT incidence of 42% (95%CI 15–72%) in a small sample of 12 ICU patients under ECMO. Furthermore, estimated VTE incidence from Mazzaccaro et al. [34] was 66%, based on a systematic CTPA and CUS screening of 32 patients admitted to medical ward. However, when excluding these three studies, the measured heterogeneity (*I*^*2*^) remained > 80% in the primary analysis.

##### Screening

Screening for VTE was performed in 14 studies [6, 14, 16, 18–20, 25, 32, 34–37, 41, 42] for DVT and three studies [16, 34, 43] for PE. Overall, studies with implemented screening found higher risks of VTE than studies without screening. In the ICU, VTE rate was 23% vs. 19% respectively; and in combined medical ward and ICU, it was 11% vs. 5% respectively (Table 3, Fig. 2b, additional figures 2b and 3b for DVT and PE separately).

**Table 3:** Meta-analytic estimates of the risk of VTE, stratified by location and screening

	VTE		DVT		PE	
	ES (95%CI)	I^**2**^	ES (95%CI)	I^**2**^	ES (95%CI)	I^**2**^
**Ward ± ICU without screening**	5% (3–9%)	84.9%	1% (0–1%)	27.9%	5% (3–8%)	83.7%
**Ward ± ICU with screening**	11% (4–22%)	93.8%	4% (1–9%)	83.3%	66% (47–81%)	–
**ICU without screening**	19% (11–28%)	91.7%	2% (0–4%)	52.4%	17% (10–26%)	90.6%
**ICU with screening**	23% (15–32%)	47.6%	20% (13–28%)	44.2%	17% (3–37%)	–

*VTE* venous thromboembolism, *DVT* deep venous thrombosis, *PE* pulmonary embolism, *ICU* intensive care unit

##### Anticoagulation

Thrombotic events where further stratified by the type of anticoagulation during hospitalization, despite a large amount of missingness. In the absence of anticoagulation, with prophylactic and with therapeutic anticoagulation, proportions of VTE were 29.4% [22, 32] (5/17), 19.8% [13, 14, 17, 18, 22, 29, 32, 35–37, 39, 40, 43] (155/781) and 7.1% [22, 29, 32, 35] (3/42), respectively (Fisher *p* = 0.047) (additional table 5).

##### Risk among medical patients only

4 studies [19, 25, 31, 35] provided data to estimate the risk of VTE after exclusion of ICU patients. Among 531 medical patients, the meta-analytic risk of VTE was 2% (95%CI 0–6%) (additional figure 4).

#### Sensitivity analyses

In 8 high-quality studies [17, 21, 27, 31, 32, 35, 37, 40], the VTE incidence was 15% (95%CI 9–23%) in the ICU only versus 7% (95%CI 2–17%) in the medical ward ± ICU (additional figure 5a). Screening was also associated with a greater incidence: 14% versus 3% in the medical ward ± ICU, and 23% versus 12% in the ICU only, with and without screening; respectively (additional figure 5b).

## Discussion

In this systematic review and meta-analysis, we extracted data from 33 studies including a total of 4009 patients. Among patients with COVID-19 hospitalized in the medical ward and/or ICU, the incidence of major VTE was 9%. The incidence of proximal lower limb DVT was 3% and the incidence of PE 8%. In ICU patients, corresponding incidences were much higher: 21, 8, and 17%, respectively. When restricting the analysis to medical patients, VTE was only found in 2%.

Previous meta-analytic efforts may have overestimated the burden of clinically relevant VTE among hospitalized patients with COVID-19. Among 30 studies published until June 24th, 2020, Porfidia et al. [44] reported an overall incidence of VTE of 26% (95%CI 1–75%), but upper extremity and distal lower limb DVT were also included. Nopp et al. [45] recently published the largest meta-analysis to date, including 66 studies with a total of 28,193 patients. Similarly to our report, they estimated an overall VTE risk of 14.1% (95%CI 11.6–16.9%), however they did not distinguish distal/proximal, and upper/lower limb DVT. Other meta-analyses have been published, but did not restrict the events to objectively diagnosed VTE, with a potential for overestimation of the risk of VTE [44–47].

The novelty of our analysis lies in the strict inclusion of objectively diagnosed clinically relevant VTE, restricted to proximal lower limb DVT and PE. Indeed, the clinical significance of distal DVT and need for anticoagulant treatment is highly debated, as its potential for embolism and recurrence is much lower than that of proximal DVT [48], without demonstrated benefit of anticoagulation in the limited randomized trials [49, 50]. The same rationale applies to catheter-related upper extremity DVT. Moreover, the diagnostic performance of CUS is lower for distal DVT than for proximal DVT, with a potential for false positive and negative findings, so that restricting the analysis to proximal DVT is likely to provide more robust data on the true DVT rate. We a priori decided to include incidental PE in the primary outcome, because the current evidence suggests that it carries the same VTE recurrence rate, and therefore clinical significance, as symptomatic PE [51]. In any case, data on symptoms of VTE were limited, being reported by 9 studies [14, 18, 20, 21, 32, 35, 37, 41, 43] for DVT, and 4 studies [16, 35, 40, 43] for PE.

Even when using these strict criteria for defining major VTE, we found an elevated risk of VTE in critically-ill COVID-19 patients (21%), despite the use of pharmacological thromboprophylaxis in most studies. This is much greater than the anticipated risk of VTE in ICU patients (5–9%) inferred from previous studies of unselected ICU patients [52, 53]. We hypothesize that, in addition to COVID-19 specific pathophysiological mechanisms such as the endothelial tropism [1] and associated coagulopathy, extrinsic factors observed in these patients such as prolonged immobility and repetitive use of muscle relaxant drugs (curare) in COVID-19 patients with acute respiratory distress syndrome (ARDS), may contribute to this increased VTE rate compared to historical ICU cohorts. Among COVID-19 patients hospitalized in general medical wards, we found a 2% risk that is in line with previous estimates of VTE [54], but this analysis excluded participants who became critically ill during their hospital stay, and likely does not apply to medical patients with severe COVID-19 at the time of admission.

The utility of VTE screening remains unknown among COVID-19 inpatients. While we found greater VTE risks in studies which included some screening, usually for DVT, we cannot infer from our results that screening is useful and should be implemented in routine practice, given the lack of comparison within individual studies. This deserves further research.

The findings of our meta-analysis call for interventional clinical trials in this setting and supports the research effort that has been launched since the beginning of the epidemic. In particular, 20 trials are comparing different levels of anticoagulation to prevent the burden of VTE and reduce the respiratory failure associated with ARDS [55]. Our group has launched the ongoing Swiss multicentric COVID-HEP trial last April, which includes both ICU patients and medical patients deemed at poor prognosis due to elevated levels of D-dimer (NCT04345848).

Strengths of this analysis include its wide search strategy, the independent selection of studies and abstraction of data, the contact with authors to request additional data and the inclusion of only VTE events with strict criteria on objective diagnosis and clinical relevance. We also acknowledge limitations. First, our search strategy was performed on June 15th, thus excluding the additional evidence published since then. However, this delay was inevitable in order to extract the outcome of interest, oftentimes with the help of multiple investigators. Second, as others, we observed a very important heterogeneity of all results, that was multifactorial. In particular, there was evident heterogeneity between study characteristics, with a mixture of inclusion criteria, use of screening and/or levels of thromboprophylaxis and different follow-up. Third, as this is a study-level meta-analysis, we were often restricted by the amount of data available in the published papers, or shared by investigators.

## Conclusions

In conclusion, this meta-analysis found that the risk of objectively confirmed clinically relevant VTE was overall 9% in patients with COVID-19 admitted to hospital. As compared to a 2% VTE risk in patients hospitalized only in a general medical ward, the risk was much higher (21%) in patients needing ICU care during their hospital stay. Such findings reinforce the need for a proper use of thromboprophylaxis in severe COVID-19 inpatients, and for interventional trials testing different thromboprophylaxis regimens.

## Supplementary Information

**Additional file 1 **Search strategies. **Table S1**. PRISMA checklist. **Table S2**. Newcastle-Ottawa scale for included cohort studies. **Table S3**. Detailed characteristics of included studies.**Table S4**. Detailed characteristics of participants. **Table S5**. VTE stratified by the type of anticoagulation (none, prophylactic, therapeutic). **Figure S1**. Funnel plot of:1a studies in medical ward ± ICU inpatients;1b studies in ICU only. **Figure S2**. Forrest plot of the estimated incidence of proximal DVT:2a stratified by medical ward and ICU;2b stratified by location and screening. **Figure S3**. Forrest plot of the estimated incidence of PE:3a stratified by general ward and ICU;3b stratified by location and screening. **Figure S4**. Forrest plot of the meta-analytic risk of VTE, restricted to medical inpatients without ICU stay. **Figure S5**. Sensitivity analysis, restricting to high-quality studies:5a stratified by location; b stratified by location and screening.

## Data Availability

All data generated or analysed during this study are included in this published article and its supplementary information files.

## Abbreviations

COVID-19: Coronavirus disease 2019
SARS-CoV2: Severe acute respiratory syndrome coronavirus 2
VTE: Venous thromboembolism
DVT: Deep vein thrombosis
CTPA: Computed tomography pulmonary angiography
PE: Pulmonary embolism
PRISMA: Preferred Reporting Items for Systematic Reviews and Meta-Analyses
CUS: Compression ultrasound
ICU: Intensive care unit
ARDS: Acute respiratory distress syndrome
