# Supplementary material for: Proximal deep vein thrombosis and pulmonary embolism in COVID-19 patients: a systematic review and meta-analysis
Source: Thromb J. 2021 Mar 9;19:15. doi: 10.1186/s12959-021-00266-x (PMC7942819; doi:10.1186/s12959-021-00266-x)
Supplement: Supplementary file 1 — Additional file 1 Search strategies. Table S1. PRISMA checklist. Table S2. Newcastle-Ottawa scale for included cohort studies. Table S3. Detailed characteristics of included studies.Table S4. Detailed characteristics of participants. Table S5. VTE stratified by the type of anticoagulation (none, prophylactic, therapeutic). Figure S1. Funnel plot of:1a studies in medical ward ± ICU inpatients;1b studies in ICU only. Figure S2. Forrest plot of the estimated incidence of proximal DVT:2a stratified by medical ward and ICU;2b stratified by location and screening. Figure S3. Forrest plot of the estimated incidence of PE:3a stratified by general ward and ICU;3b stratified by location and screening. Figure S4. Forrest plot of the meta-analytic risk of VTE, restricted to medical inpatients without ICU stay. Figure S5. Sensitivity analysis, restricting to high-quality studies:5a stratified by location; b stratified by location and screening. [file 12959_2021_266_MOESM1_ESM.docx]

**ADDITIONAL FILES**

Table of contents

[Additional file 1. Search strategies 3](#_Toc54865860)

[Additional table 1. PRISMA checklist. 5](#_Toc54865869)

[Additional table 2. Newcastle-Ottawa scale for included cohort studies. 7](#_Toc54865870)

[Additional table 3. Detailed characteristics of included studies. 9](#_Toc54865883)

[Additional table 4. Detailed characteristics of participants. 12](#_Toc54865884)

[Additional table 5. VTE stratified by the type of anticoagulation (none, prophylactic, therapeutic). 15](#_Toc54865885)

[Additional figure 1. Funnel plot of: 16](#_Toc54865886)

[1a studies in medical ward ± ICU inpatients; 16](#_Toc54865887)

[1b studies in ICU only. 17](#_Toc54865888)

[Additional figure 2. Forrest plot of the estimated incidence of proximal DVT: 18](#_Toc54865889)

[2a stratified by medical ward and ICU; 18](#_Toc54865890)

[2b stratified by location and screening. 19](#_Toc54865891)

[Additional figure 3. Forrest plot of the estimated incidence of PE: 20](#_Toc54865892)

[3a stratified by general ward and ICU; 20](#_Toc54865893)

[3b stratified by location and screening. 21](#_Toc54865894)

[Additional figure 4. Forrest plot of the meta-analytic risk of VTE, restricted to medical inpatients without ICU stay. 22](#_Toc54865895)

[Additional figure 5. Sensitivity analysis, restricting to high-quality studies: 23](#_Toc54865896)

[5a stratified by location ; 23](#_Toc54865897)

[5b stratified by location and screening. 23](#_Toc54865898)

Additional file 1. Search strategies

**Search equation Embase**

('coronavirus disease 2019'/de OR 'coronavirus disease 2019':ab,ti OR 'covid 19'/de OR 'covid 19':ab,ti OR 'covid':ab,ti OR 'covid19':ab,ti OR 'severe acute respiratory syndrome coronavirus 2'/de OR 'severe acute respiratory syndrome coronavirus 2':ab,ti OR 'sars-related coronavirus'/de OR 'sars-related coronavirus':ab,ti OR 'sars-like cov':ab,ti OR 'sars-related cov':ab,ti OR 'sars coronavirus 2'/de OR 'sars coronavirus 2':ab,ti OR 'sars virus':ab,ti OR 'hcov-sars':ab,ti OR 'sars-cov':ab,ti OR 'sars-cov-2':ab,ti OR 'coronavirus':ab,ti OR 'coronavirinae'/de OR 'coronavirinae':ab,ti OR 'coronaviridae'/de OR 'coronaviridae':ab,ti OR 'coronaviridae infection'/de OR 'coronaviridae infection':ab,ti OR 'ncov':ab,ti OR '2019-ncov':ab,ti OR 'ncov-2019':ab,ti OR 'wuhan seafood market pneumonia virus':ab,ti) AND ('thrombosis'/de OR 'thrombosis':ab,ti OR 'thromboses':ab,ti OR 'thrombus':ab,ti OR 'blood clot':ab,ti OR 'blood clots':ab,ti OR 'vein thrombosis'/de OR 'vein thrombosis':ab,ti OR 'venous thrombosis':ab,ti OR 'deep vein thrombosis'/de OR 'deep vein thrombosis':ab,ti OR 'thrombotic':ab,ti OR 'thromboembolism'/de OR thromboembolism:ab,ti OR 'thromboembolisms':ab,ti OR 'thromboembolic':ab,ti OR 'thromboemboly':ab,ti OR 'embolism':ab,ti OR 'embolus':ab,ti OR ('embolism':ab,ti AND 'thrombosis':ab,ti) OR 'leg thrombosis'/de OR 'leg thrombosis':ab,ti OR 'vein embolism'/de OR 'vein embolism':ab,ti OR 'venous thromboembolism'/de OR 'venous thromboembolism':ab,ti OR 'lung embolism'/de OR 'lung embolism':ab,ti OR 'lung emboly':ab,ti OR 'pulmonary embolism':ab,ti OR 'pulmonary embolus':ab,ti OR 'pe':ab,ti OR 'dvt':ab,ti OR 'vte':ab,ti) AND [2019-2020]/py AND [english]/lim

Last search carried out on June 15th, 2020: 287 hits

**Search equation PubMed**

("Coronavirus"[Mesh] OR "COVID-19" [Supplementary Concept] OR "SARS Virus"[Mesh] OR "severe acute respiratory syndrome coronavirus 2" [Supplementary Concept] OR "coronavirus disease 2019" OR "COVID-19" OR "COVID 19" OR "COVID" OR "COVID19" OR "severe acute respiratory syndrome coronavirus 2" OR "SARS-related coronavirus" OR "SARS-like CoV" OR "SARS-related CoV" OR "SARS coronavirus 2" OR "SARS Virus" OR "SARS Virus" OR "HCoV-SARS" OR "SARS-CoV" OR "SARS-CoV-2" OR "coronavirus" OR "coronavirinae" OR "coronaviridae" OR "coronaviridae infection" OR "nCoV" OR "2019-nCoV" OR "nCoV-2019" OR "Wuhan seafood market pneumonia virus") AND ("Thrombosis"[Mesh] OR "Venous Thrombosis"[Mesh] OR "Thromboembolism"[Mesh] OR "Embolism"[Mesh] OR "Embolism and Thrombosis"[Mesh] OR "Pulmonary Embolism"[Mesh] OR "Venous Thromboembolism"[Mesh] OR "thrombosis" OR "thromboses" OR "thrombus" OR "blood clot" OR "blood clots" OR "vein thrombosis" OR "venous thrombosis" OR "deep vein thrombosis" OR "thrombotic" OR "thromboembolism" OR "thromboembolisms" OR "thromboembolic" OR "thromboemboly" OR "embolism" OR "embolus" OR "embolism and thrombosis" OR "leg thrombosis" OR "vein embolism" OR "venous thromboembolism" OR "lung embolism" OR "lung emboly" OR "pulmonary embolism" OR "pulmonary embolus" OR "PE" OR "DVT" OR "VTE")

Filters: English, Timespan=2019-2020

Last search carried out on June 15th, 2020: 505 hits

**Search equation Cochrane Library Wiley**

("coronavirus disease 2019":ab,ti OR "covid 19":ab,ti OR "covid":ab,ti OR "covid19":ab,ti OR "severe acute respiratory syndrome coronavirus 2":ab,ti OR "sars-related coronavirus":ab,ti OR "sars-like cov":ab,ti OR "sars-related cov":ab,ti OR "sars coronavirus 2":ab,ti OR "sars virus":ab,ti OR "hcov-sars":ab,ti OR "sars-cov":ab,ti OR "sars-cov-2":ab,ti OR "coronavirus":ab,ti OR "coronavirinae":ab,ti OR "coronaviridae":ab,ti OR "coronaviridae infection":ab,ti OR "ncov":ab,ti OR "2019-ncov":ab,ti OR "ncov-2019":ab,ti OR "wuhan seafood market pneumonia virus":ab,ti) AND ("thrombosis":ab,ti OR "thromboses":ab,ti OR "thrombus":ab,ti OR "blood clot":ab,ti OR "blood clots":ab,ti OR "vein thrombosis":ab,ti OR "venous thrombosis":ab,ti OR "deep vein thrombosis":ab,ti OR "thrombotic":ab,ti OR "thromboembolism":ab,ti OR "thromboembolisms":ab,ti OR "thromboembolic":ab,ti OR "thromboemboly":ab,ti OR "embolism":ab,ti OR "embolus":ab,ti OR "leg thrombosis":ab,ti OR "vein embolism":ab,ti OR "venous embolism":ab,ti OR "venous thromboembolism":ab,ti OR "lung embolism":ab,ti OR "lung emboly":ab,ti OR "pulmonary embolism":ab,ti OR "pulmonary embolus":ab,ti OR "pe":ab,ti OR "dvt":ab,ti OR "vte":ab,ti)

Custom range 2019-2020

Last search carried out on June 15th, 2020: 15 hits

**Search equation Web of Science Core collection**

(ALL=("coronavirus disease 2019" OR "covid 19" OR "covid" OR "covid19" OR "severe acute respiratory syndrome coronavirus 2" OR "sars-related coronavirus" OR "sars-like cov" OR "sars-related cov" OR "sars coronavirus 2" OR "sars virus" OR "hcov-sars" OR "sars-cov" OR "sars-cov-2" OR "coronavirus" OR "coronavirinae" OR "coronaviridae" OR "coronaviridae infection" OR "ncov" OR "2019-ncov" OR "ncov-2019" OR "wuhan seafood market pneumonia virus")) AND TS=(("thrombosis" OR "thromboses" OR "thrombus" OR "blood clot" OR "blood clots" OR "vein thrombosis" OR "venous thrombosis" OR "deep vein thrombosis" OR "thrombotic" OR "thromboembolism" OR "thromboembolisms" OR "thromboembolic" OR "thromboemboly" OR "embolism" OR "embolus" OR "leg thrombosis" OR "vein embolism" OR "venous embolism" OR "venous thromboembolism" OR "lung embolism" OR "lung emboly" OR "pulmonary embolism" OR "pulmonary embolus" OR "pe" OR "dvt" OR "vte")) AND LANGUAGE: (English), Timespan=2019-2020

Last search carried out on June 15th, 2020: 83 hits

# Additional table 1. PRISMA checklist.

| **Section/topic** | **#** | **Checklist item** | **Reported on page #** |
| --- | --- | --- | --- |
| **TITLE** | | |  |
| Title | 1 | Identify the report as a systematic review, meta-analysis, or both. | 1 |
| **ABSTRACT** | | |  |
| Structured summary | 2 | Provide a structured summary including, as applicable: background; objectives; data sources; study eligibility criteria, participants, and interventions; study appraisal and synthesis methods; results; limitations; conclusions and implications of key findings; systematic review registration number. | 3-4 |
| **INTRODUCTION** | | |  |
| Rationale | 3 | Describe the rationale for the review in the context of what is already known. | 5 |
| Objectives | 4 | Provide an explicit statement of questions being addressed with reference to participants, interventions, comparisons, outcomes, and study design (PICOS). | 5 |
| **METHODS** | | |  |
| Protocol and registration | 5 | Indicate if a review protocol exists, if and where it can be accessed (e.g., Web address), and, if available, provide registration information including registration number. | 6 |
| Eligibility criteria | 6 | Specify study characteristics (e.g., PICOS, length of follow-up) and report characteristics (e.g., years considered, language, publication status) used as criteria for eligibility, giving rationale. | 6 |
| Information sources | 7 | Describe all information sources (e.g., databases with dates of coverage, contact with study authors to identify additional studies) in the search and date last searched. | 6 |
| Search | 8 | Present full electronic search strategy for at least one database, including any limits used, such that it could be repeated. | 6 |
| Study selection | 9 | State the process for selecting studies (i.e., screening, eligibility, included in systematic review, and, if applicable, included in the meta-analysis). | 6 |
| Data collection process | 10 | Describe method of data extraction from reports (e.g., piloted forms, independently, in duplicate) and any processes for obtaining and confirming data from investigators. | 7 |
| Data items | 11 | List and define all variables for which data were sought (e.g., PICOS, funding sources) and any assumptions and simplifications made. | 6-7 |
| Risk of bias in individual studies | 12 | Describe methods used for assessing risk of bias of individual studies (including specification of whether this was done at the study or outcome level), and how this information is to be used in any data synthesis. | 7 |
| Summary measures | 13 | State the principal summary measures (e.g., risk ratio, difference in means). | 8 |
| Synthesis of results | 14 | Describe the methods of handling data and combining results of studies, if done, including measures of consistency (e.g., I^2^) for each meta-analysis. | 8 |

| **Section/topic** | **#** | **Checklist item** | **Reported on page #** |
| --- | --- | --- | --- |
| Risk of bias across studies | 15 | Specify any assessment of risk of bias that may affect the cumulative evidence (e.g., publication bias, selective reporting within studies). | 7-8 |
| Additional analyses | 16 | Describe methods of additional analyses (e.g., sensitivity or subgroup analyses, meta-regression), if done, indicating which were pre-specified. | 8 |
| **RESULTS** | | |  |
| Study selection | 17 | Give numbers of studies screened, assessed for eligibility, and included in the review, with reasons for exclusions at each stage, ideally with a flow diagram. | 9 |
| Study characteristics | 18 | For each study, present characteristics for which data were extracted (e.g., study size, PICOS, follow-up period) and provide the citations. | 9 |
| Risk of bias within studies | 19 | Present data on risk of bias of each study and, if available, any outcome level assessment (see item 12). | 10 |
| Results of individual studies | 20 | For all outcomes considered (benefits or harms), present, for each study: (a) simple summary data for each intervention group (b) effect estimates and confidence intervals, ideally with a forest plot. | 10-13 |
| Synthesis of results | 21 | Present results of each meta-analysis done, including confidence intervals and measures of consistency. | 10-13 |
| Risk of bias across studies | 22 | Present results of any assessment of risk of bias across studies (see Item 15). | 10 |
| Additional analysis | 23 | Give results of additional analyses, if done (e.g., sensitivity or subgroup analyses, meta-regression [see Item 16]). | 11-13 |
| **DISCUSSION** | | |  |
| Summary of evidence | 24 | Summarize the main findings including the strength of evidence for each main outcome; consider their relevance to key groups (e.g., healthcare providers, users, and policy makers). | 14 |
| Limitations | 25 | Discuss limitations at study and outcome level (e.g., risk of bias), and at review-level (e.g., incomplete retrieval of identified research, reporting bias). | 16 |
| Conclusions | 26 | Provide a general interpretation of the results in the context of other evidence, and implications for future research. | 17 |
| **FUNDING** | | |  |
| Funding | 27 | Describe sources of funding for the systematic review and other support (e.g., supply of data); role of funders for the systematic review. | 2 |

# Additional table 2. Newcastle-Ottawa scale for included cohort studies.

| Study (first author) | Selection | | | | Comparability | | Outcome | | | Total 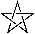 |
| --- | --- | --- | --- | --- | --- | --- | --- | --- | --- | --- |
| Al-Samkari *et al.* | 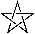 | NA | 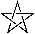 |  | NA | NA | 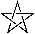 |  |  | 3 |
| Annuziata *et al.* |  | NA |  |  | NA | NA | 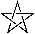 |  |  | 1 |
| Artifoni *et al.* | 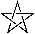 | NA | 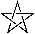 |  | NA | NA | 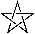 |  | 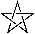 | 4 |
| Beun *et al.* | 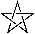 | NA |  |  | NA | NA | 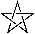 |  |  | 2 |
| Beyls *et al.* |  | NA |  |  | NA | NA | 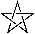 |  |  | 1 |
| Campochiaro *et al.* | 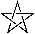 | NA | 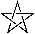 |  | NA | NA | 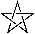 | 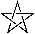 | 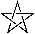 | 5 |
| Cattaneo *et al.* | 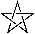 | NA |  |  | NA | NA | 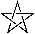 |  |  | 2 |
| Criel *et al.* | 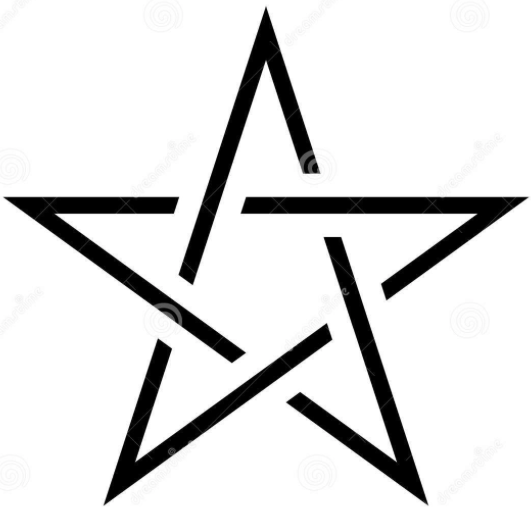 | NA |  |  | NA | NA | 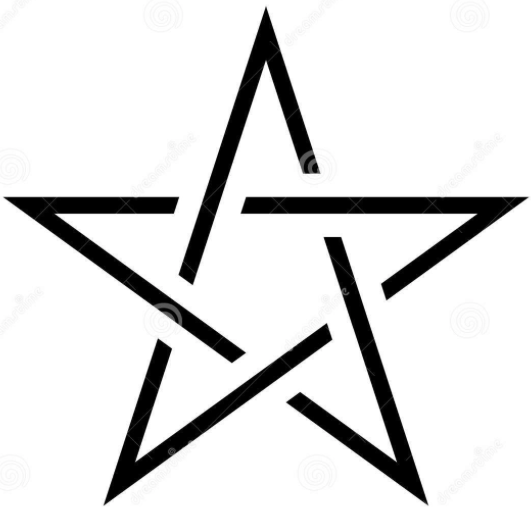 |  |  | 2 |
| Demelo-Rodriguez *et al.* |  | NA | 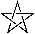 |  | NA | NA | 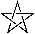 |  | 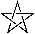 | 3 |
| Desborough *et al.* | 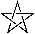 | NA | 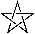 |  | NA | NA | 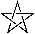 | 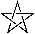 |  | 4 |
| Faggiano *et al.* | 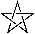 | NA |  |  | NA | NA | 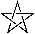 | 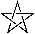 |  | 3 |
| Fraisse *et al.* | 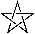 | NA |  |  | NA | NA | 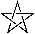 |  | 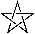 | 3 |
| Galeano-Valle *et al.* | 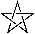 | NA | 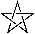 |  | NA | NA | 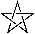 |  |  | 3 |
| Grandmaison *et al.* | 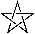 | NA | 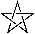 |  | NA | NA | 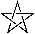 |  |  | 3 |
| Grillet *et al.* | 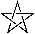 | NA |  |  | NA | NA | 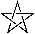 |  |  | 2 |
| Helms *et al.* | 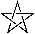 | NA | 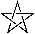 |  | NA | NA | 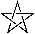 | 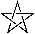 | 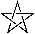 | 5 |
| Hippensteel *et al.* | 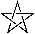 | NA | 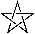 |  | NA | NA | 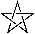 |  |  | 3 |
| Klok *et al.* | 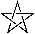 | NA |  |  | NA | NA | 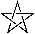 | 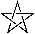 | 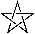 | 4 |
| Llitjos *et al.* | 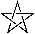 | NA | 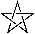 |  | NA | NA | 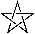 |  |  | 3 |
| Lodigiani *et al.* | 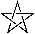 | NA | 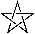 |  | NA | NA | 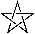 | 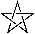 |  | 4 |
| Longchamp *et al.* | 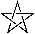 | NA | 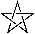 |  | NA | NA | 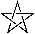 | 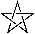 |  | 4 |
| Maatman *et al.* | 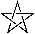 | NA | 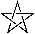 |  | NA | NA | 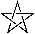 |  | 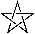 | 4 |
| Mazzaccaro *et al.* | 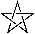 | NA | 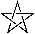 |  | NA | NA | 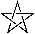 |  |  | 3 |
| Middeldorp *et al.* | 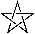 | NA | 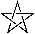 |  | NA | NA | 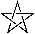 | 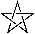 | 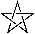 | 5 |
| Nahum *et al.* | 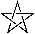 | NA | 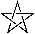 |  | NA | NA | 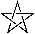 |  |  | 3 |
| Pavoni *et al.* | 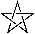 | NA | 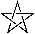 |  | NA | NA | 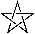 | 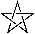 | 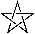 | 5 |
| Poissy *et al.* | 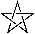 | NA | 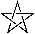 |  | NA | NA | 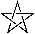 |  |  | 3 |
| Ren *et al.* | 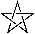 | NA |  |  | NA | NA | 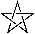 |  |  | 2 |
| Tavazzi *et al.* | 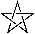 | NA |  |  | NA | NA | 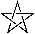 |  |  | 2 |
| Thomas *et al.* | 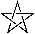 | NA | 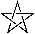 |  | NA | NA | 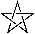 | 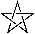 |  | 4 |
| Voicu *et al.* | 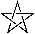 | NA |  |  | NA | NA | 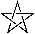 |  | 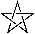 | 3 |
| Zhang *et al.* | 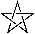 | NA |  |  | NA | NA |  |  |  | 2 |
| Zotzmann *et al.* |  | NA |  |  | NA | NA |  |  |  | 2 |

NA = not applicable, because the study lack of a non-exposed cohort.

**Newcastle-Ottawa quality assessment scale for cohort studies**

**Selection**

1) Representativeness of the exposed cohort

a) bicentric cohort from the medical ward or intensive care unit

b) monocentric cohort from the medical ward or intensive care unit

c) selected group of users e.g. elevated D-dimer, disseminated intravascular coagulation

d) no description of the derivation of the cohort

2) Selection of the non exposed cohort

a) drawn from the same community as the exposed cohort

b) drawn from a different source

c) no description of the derivation of the non exposed cohort

3) Ascertainment of exposure

a) 100% PCR positivity for COVID-19

b) 80-99% PCR positivity for COVID-19 and/or diagnostic based on CTPA imaging

c) < 80% PCR positivity for COVID-19 or diagnostic based on clinical suspicion only

d) no description

4) Demonstration that outcome of interest was not present at start of study

a) yes

b) no

**Comparability**

1) Comparability of cohorts on the basis of the design or analysis

a) study controls for age

b) study controls for sex

**Outcome**

1) Assessment of outcome

a) independent blind assessment

b) record linkage

c) self report

d) no description

2) Was follow-up long enough, defined as minimum 7 days, for outcomes to occur

a) yes

b) no

3) Adequacy of follow up of cohorts

a) complete follow up - all subjects accounted for

b) subjects lost to follow up unlikely to introduce bias - small number lost - > 80 % follow up, or description provided of those lost)

c) follow up rate < 80% (select an adequate %) and no description of those lost

d) no statement

Note: A study can be awarded a maximum of one star for each numbered item within the Selection and Outcome categories. A maximum of two stars can be given for Comparability

Reference: Wells GA Shea B O'Connell D Peterson J Welch V Losos M et al. The Newcastle-Ottawa Scale (NOS) for assessing the quality of non randomized studies in meta-analysis; 2009.
Available at http://www.ohri.ca/programs/clinical_epidemiology/oxford.asp

# Additional table 3. Detailed characteristics of included studies.

| **Study** | **Journal** | **Country** | **Design** | | **Study period** (DD.MM-DD.MM-YY) | **VTE risk** | **Sample size**, n | **PCR positivity,** n (%) | **ICU admission,** n | **SOFA score,** median or mean | **MV,** n | **HD,** n | **ECMO,** n | **Vaso.,** n | **Anticoagulation during hospitalization,** n | | |
| --- | --- | --- | --- | --- | --- | --- | --- | --- | --- | --- | --- | --- | --- | --- | --- | --- | --- |
|  |  |  | **ROC, POC, CS** | **Mono. or multi.** |  |  |  |  |  |  |  |  |  |  | **None** | **Proph.** | **Th.** |
| Al-Samkari *et al.* | Blood | USA | ROC | multi | 01.03-08.04.20 | 1 | 400 | 400 (100) | - | - | 144 | - | - | - | 11 | 355 | 35 |
| Annunziata *et al.* | Turkish thoracic journal | Italy | ROC | mono | spring 2020 | 4 | 21 | - | 21 | - | - | - | - | - | 0 | 21 | 0 |
| Artifoni *et al.* | Journal of thrombosis and thrombolysis | France | ROC | multi | 25.03-10.04.20 | 1 | 71 | - | 13 | 1 | 8 | - | - | - | - | 70 | - |
| Beun *et al.* | International journal of laboratory hematology | Netherlands | ROC | mono | 16.03-09.04.20 | 3 | 75 | - | 75 | - | - | - | - | - | - | - | - |
| Beyls *et al.* | British journal of anaesthesia | France | POC | mono | 01.03-04.04.20 | 4 | 12 | - | 12 | 11 | 12 | - | 12 | - | - | - | - |
| Campochiaro *et al.* | European journal of internal medicine | Italy | ROC | mono | spring 2020 | 1 | 65 | 65 (100) | 35 | - | - | - | - | - | - | 65 | 0 |
| Cattaneo *et al.* | Thrombosis and haemostasis | Italy | POC | mono | until 14.04.20 | 1 | 64 | - | - | - | - | - | - | - | - | 64 | - |
| Criel *et al.* | European respiratory journal | Belgium | CS | mono | 03.04-22.04.20 | 1 | 82 | - | 30 | - | 21 | - | 0 | - | 3 | 78 | - |
| Demelo-Rodriguez *et al.* | Thrombosis research | Spain | POC | mono | from the first half of April 2020 | 2 | 156 | 133 (85.3) | 16 | - | - | - | - | - | 3 | 153 | 0 |
| Desborough *et al.* | Thrombosis research | UK | ROC | mono | 01.03-31.03.20 | 3 | 66 | 66 (100) | 66 | - | 52 | 18 | 8 | 31 | 0 | 54 | 12 |
| Faggiano *et al.* | International journal of cardiology | Italy | ROC | mono | spring 2020 | 1 | 25 | - | - | - | - | - | - | - | - | 5 | 4 |
| Fraisse *et al.* | Critical care | France | ROC | mono | 06.03-06.05.20 | 3 | 92 | - | 92 | 4 | 82 | 22 | - | 57 | 0 | - | 49 |
| Galeano-Valle *et al.* | Thrombosis research | Spain | POC | mono | 26.03-15.04.20 | 1 | 785 | - | - | - | - | - | - | - | - | - | - |
| Grandmaison *et al.* | Thrombosis and haemostasis | Switzerland | POC | mono | spring 2020 | 1 | 58 | 58 (100) | 29 | - | 14 | - | - | - | - | - | - |
| Grillet *et al.* | Radiology | France | ROC | mono | 15.03-14.04.20 | 1 | 280 | - | - | - | - | - | - | - | - | - | - |
| Helms *et al.* | Intensive care medicine | France | POC | multi | 03.03-07.04.20 | 3 | 150 | 150 (100) | 150 | 8 | 150 | 29 | 12 | - | 0 | 105 | 45 |
| Hippensteel *et al.* | British journal of haemotology | USA | ROC | mono | 18.03-06.05.20 | 3 | 91 | 91 (100) | 91 | - | 77 | - | 0 | 61 | - | - | - |
| Klok *et al.* | Thrombosis research | Netherlands | ROC | multi | 07.03-22.04.20 | 3 | 184 | - | 184 | - | - | - | - | - | - | 167 | 17 |
| Llitjos *et al.* | Journal of thrombosis and haemostasis | France | ROC | multi | 19.03-11.04.20 | 3 | 26 | 26 (100) | 26 | 3 | 26 | 4 | 2 | 23 | - | 8 | 18 |
| Lodigiani *et al.* | Thrombosis research | Italy | ROC | mono | 13.02-10.04.20 | 1 | 388 | 388 (100) | 61 | - | - | - | - | - | - | - | - |
| Longchamp *et al.* | Research and practice in thrombosis and haemostasis | Switzerland | POC | mono | 08.03-09.05.20 | 3 | 25 | 25 (100) | 25 | - | 23 | - | 1 | 19 | 1 | 22 | 2 |
| Maatman *et al.* | Critical care medicine journal | USA | ROC | multi | 12.03-06.05.20 | 3 | 109 | 109 (100) | 109 | - | 103 | 16 | - | 70 | - | 103 | 4 |
| Mazzaccaro *et al.* | Journal of clinical medicine | Italy | ROC | mono | 18.03-20.04.20 | 1 | 32 | 32 (100) | 2 | 2.8 | - | - | - | - | - | - | - |
| Middeldorp *et al.* | Journal of thrombosis and haemostasis | Netherlands | ROC | mono | 02.03-30.04.20 | 1 | 198 | 173 (87.4) | 75 | - | 75 | - | - | - | - | 167 | 19 |
| Nahum *et al.* | Journal of the American medical association network | France | POC | mono | mid March - beginning April 2020 | 3 | 34 | 26 (76.5) | 34 | - | 34 | - | 4 | 26 | 0 | 34 | 0 |
| Pavoni *et al.* | Journal of thrombosis and thrombolysis | Italy | ROC | mono | 28.02-10.04.20 | 3 | 40 | 40 (100) | 40 | 4 | 4 | - | - | - | - | 40 | - |
| Poissy *et al.* | Circulation AHA | France | ROC | mono | 27.02-09.04.20 | 3 | 107 | 107 (100) | 107 | - | 67 | - | - | - | - | - | - |
| Ren *et al.* | Circulation AHA | China | CS | multi | 29.02-02.03.20 | 3 | 48 | 48 (100) | 48 | - | 18 | - | - | - | 1 | 47 | 0 |
| Tavazzi *et al.* | Intensive care medicine | Italy | POC | mono | from 21.02.20 | 3 | 54 | - | 54 | - | 54 | - | - | - | - | 54 | - |
| Thomas *et al.* | Thrombosis research | UK | ROC | mono | 15.03-14.04.20 | 3 | 62 | 62 (100) | 62 | - | 52 | 23 | - | - | 0 | 62 | - |
| Voicu *et al.* | Journal of the American college of cardiology | France | POC | mono | 13.03-03.04.20 | 3 | 56 | - | 56 | - | 56 | - | 5 | 18 | 0 | 49 | 7 |
| Zhang *et al.* | Circulation AHA | China | POC | mono | 29.01-24.03.20 | 1 | 143 | - | 15 | - | 23 | - | - | - | 90 | 53 | 0 |
| Zotzmann *et al.* | Intensive care medicine | Germany | POC | mono | spring 2020 | 4 | 10 | 10 (100) | 10 | - | 10 | - | - | 3 | 0 | 10 | 0 |

ROC = retrospective observational cohort, POC = prospective observational cohort, CS = cross-sectionnal, mono. = monocentric, multi. = multicentric VTE = venous thromboembolism, PCR = polymerase chain reaction, ICU = intensive care unit, SOFA = sequential organ failure assessment, MV = mechanical ventilation, HD = hemodialysis, ECMO = extracorporeal membrane oxygenation, Vaso. = vasoconstrictor, Proph. = prohylactic, Th. = therapeutic.

VTE risk: 1 (low) corresponds to medical ward admission; 2 (moderate) corresponds to medical ward admission with an additional risk factor i.e. elevated D-dimer, disseminated intravascular coagulation; 3 (high) corresponds to ICU admission; 4 (very high) corresponds to ICU admission with an additional risk factor.

# Additional table 4. Detailed characteristics of participants.

| **Study** | **Characteristics** | | | | | | | | | | | | **Laboratory data** | | | | | |
| --- | --- | --- | --- | --- | --- | --- | --- | --- | --- | --- | --- | --- | --- | --- | --- | --- | --- | --- |
|  | **M/F** | **Age (years),** mean or median | **CVD,** n | **HTN,** n | **DM,** n | **CKD,** n | **CRD,** n | **BMI (kg/m^2^),** mean or median | **Cancer,** n | **History of VTE,** n | **Surgery <3 mo,** n | **Smoking,** n | **Creatinemia (mg/dL),** mean (±SD) or median (IQR) | **Platelet (*10^9^/L),** mean (±SD) or median (IQR) | **aPTT (sec),** mean (±SD) or median (IQR) | **PT,** mean (±SD) or median (IQR) | **Fibrinogen (g/L),** mean (±SD) or median (IQR) | **D-dimer (µg/L),** mean (±SD) or median (IQR) |
| Al-Samkari *et al.* | 228/172 | 61.8 | - | - | 123 | 10 | 78 | - | - | - | - | - | - | - | - | - | - | - |
| Annunziata *et al.* | - | - | - | - | - | - | - | - | - | - | - | - | - | - | - | - | - | - |
| Artifoni *et al.* | 43/28 | 64 | - | 29 | 14 | - | - | 27.3 | 4 | 5 | 7 | 6 | 0.9 (0.8-1.0) | 212 (162-248) | - | - | 4.9 (4.3-6.5) | 790 (480-1610) |
| Beun *et al.* | - | - | - | - | - | - | - | - | - | - | - | - | - | - | - | - | - | - |
| Beyls *et al.* | 10/2 | 62 | - | - | - | - | - | 29.5 | - | - | - | - | - | 240 (151-329) | - | 60 % (52-70) | 7 (6-9) | 8300 (4700-24000) |
| Campochiaro *et al.* | 56/9 | - | 10 | 28 | 10 | 8 | 3 | - | 3 | - | - | 2 | - | - | - | - | - | - |
| Cattaneo *et al.* | 35/29 | 70 | - | - | - | - | - | - | 7 | 0 | - | - | - | 286 (222-384) | - | - | 4.8 (3.9-5.4) | 458 (252-903) |
| Criel *et al.* | 48/34 | 63.9 | - | 30 | 14 | - | - | 30 | - | - | - | - | - | - | - | - | - | 1300 |
| Demelo-Rodriguez *et al.* | 102/54 | 68.1 | - | - | - | - | - | 26.9 | 16 | 2 | - | - | - | 264.5 (132.4) | - | - | - | 2148 (1532-4002) |
| Desborough *et al.* | 48/18 | 59 | 7 | 30 | 27 | 9 | 6 | 28 | 5 | 5 | - | - | - | 207 (154-272) | - | - | 6.5 (4.6-7.5) | 2400 (1100-26200) |
| Faggiano *et al.* | 21/4 | 71.2 | 13 | 8 | 2 | - | - | - | 5 | - | - | - | - | - | - | - | - | 2270.6 |
| Fraisse *et al.* | 73/19 | 61 | 9 | 59 | 35 | 7 | 18 | 30 | - | 5 | - | - | - | 227 (182-307) | - | 86 %, (76-96) | 7.8 (6.1-8.8) | 2400 (1700-7900) |
| Galeano-Valle *et al.* | - | - | - | - | - | - | - | - | - | - | - | - | - | - | - | - | - | - |
| Grandmaison *et al.* | - | - | - | - | - | - | - | - | - | - | - | - | - | - | - | - | - | - |
| Grillet *et al.* | - | - | - | - | - | - | - | - | - | - | - | - | - | - | - | - | - | - |
| Helms *et al.* | 122/28 | 63 | 72 | - | 30 | 6 | 21 | - | 9 | 8 | - | - | - | 200 (152-267) | - | 84 % (73-91) | 7 (6.1-7.7) | 2270 (1160-20000) |
| Hippensteel *et al.* | 53/38 | 56.5 | 20 | - | 28 | 11 | 17 | 32.4 | 3 | - | - | 16 | 1.6 | 200 | - | - | - | - |
| Klok *et al.* | 139/45 | 64 | - | - | - | - | - | - | 5 | - | - | - |  | - | - | - | - | - |
| Llitjos *et al.* | 20/6 | 68 | - | 22 | - | - | - | 30.2 | 0 | 1 | 1 | 7 | 0.9 (0.6-1.1) | 234 (169-306) | - | - | 7 (6.4-7.4) | 1750 (1130-2850) |
| Lodigiani *et al.* | 264/124 | 66 | 54 | 183 | 88 | 61 | 35 | - | 25 | 12 | - | 45 | - | - | - | - | - | - |
| Longchamp *et al.* | 16/9 | 68 | 3 | 10 | 1 | - | 2 | 27.5 | 2 | 0 | - | 6 | 0.8 (0.7-1.1)^∆^ | - | - | - | 6.4 (4.5-7.2)^∆^ | 2071 (953-3606)^∆^ |
| Maatman *et al.* | 62/47 | 61 | - | 74 | 43 | 16 | 18 | 34.8 | - | - | - | 33 | 1.2 (0.9-1.9) | 207 (152-255) | - | - | 5.4 (4.4-6.5) | 506 (321-973) |
| Mazzaccaro *et al.* | 23/9 | 68.6 | 7 | 20 | 7 | - | 1 | 27.1 | - | - | - | 14 | 0.9 (0.3) | 186.5 (83.4) | 34.3 (4.7) | 14.5 sec (3.9) | 5.9 (1.4) | 3700 (4800) |
| Middeldorp *et al.* | 130/68 | 61 | - | - | - | - | - | 27 | 7 | 11 | - | - | - | 239 (93) | - | - | - | 1100 (700-2300) |
| Nahum *et al.* | 25/9 | 62.2 | 3 | 13 | 15 | - | 2 | 31.4 | 1 | - | - | - | 1 (0.5) | 256 (107) | - | 85 % (11.4) | 7.6 (1.7) | 5100 |
| Pavoni *et al.* | 24/16 | 61 | 12 | 16 | 16 | - | 4 | 28.4 | - | - | - | - | - | 317.5 (168) | 32.2 (2.9) | 65.1 % (9.8) | 9.0 (1.1) | 1556 (1090) |
| Poissy *et al.* | - | - | - | - | - | - | - | - | - | - | - | - | - | - | - | - | - | - |
| Ren *et al.* | 26/22 | 70 | 11 | 19 | 13 | - | - | - | - | 0 | 0 | - | - | - | - | - | 4.1 (3.5-4.6) | 3480 (830-9230) |
| Tavazzi *et al.* | - | - | - | - | - | - | - | - | - | - | - | - | - | - | - | - | - | - |
| Thomas *et al.* | 43/19 | 59 | - | - | - | - | - | - | 1 | 1 | - | - | - | - | - | - | - | 394 (122-3627)^∆^ |
| Voicu *et al.* | 42/14 | - | 11 | 26 | 25 | - | - | - | - | 0 | - | - | - | - | - | - | 7.5 | - |
| Zhang *et al.* | 74/69 | 63 | 17 | 56 | 26 | 4 | - | 23.6 | 7 | 1 | - | 9 | 0.7 (0.6-0.9) | 221 (152.3-287.5) | 34.8 (30.8-39.3) | 13.6 sec (12.6-14.8) | - | 2700 (600-8000) |
| Zotzmann *et al.* | - | - | - | - | - | - | - | - | - | - | - | - | - | - | - | - | - | - |

M/F = male/female, CVD = cardiovascular disease, HTN = hypertension, DM = diabetes mellitus, CKD = chronic kidney disease, CRD = chronic respiratory disease, BMI = body mass index, VTE = venous thromboembolism, mo = months, SD = standard deviation, IQR = interquartile range, aPPT = activated partial thromboplastin time, sec = second, PT = prothrombin time.

- = not available, ^∆^ expressed as median (range).

# Additional table 5. VTE stratified by the type of anticoagulation (none, prophylactic, therapeutic).

| **Study** | **Sample size, n** | **Total VTE, n** | **No anticoagulation, n** | | | **Prophylactic anticoagulation, n** | | | **Therapeutic anticoagulation, n** | | |
| --- | --- | --- | --- | --- | --- | --- | --- | --- | --- | --- | --- |
|  |  |  | **Total** | **VTE** | **No VTE** | **Total** | **VTE** | **No VTE** | **Total** | **VTE** | **No VTE** |
| Al-Samkari *et al.* | 400 | 14 | 11 | - | - | 355 | - | - | 35 | - | - |
| Annunziata *et al.* | 21 | 17 | 0 | 0 | 0 | 21 | 17 | 4 | 0 | 0 | 0 |
| Artifoni *et al.* | 71 | 7 | - | - | - | 70 | 7 | 63 | - | - | - |
| Beun *et al.* | 75 | 20 | - | - | - | - | - | - | - | - | - |
| Beyls *et al.* | 12 | 6 | - | - | - | - | - | - | - | - | - |
| Campochiaro *et al.* | 65 | 5 | 0 | 0 | 0 | 65 | 5 | 60 | 0 | 0 | 0 |
| Cattaneo *et al.* | 64 | 0 | 0 | 0 | 0 | 64 | 0 | 64 | 0 | 0 | 0 |
| Criel *et al.* | 82 | 3 | 3 | - | - | 79 | - | - | 0 | 0 | 0 |
| Demelo-Rodriguez *et al.* | 156 | 1 | 3 | - | - | 153 | - | - | 0 | 0 | 0 |
| Desborough *et al.* | 66 | 6 | 0 | 0 | 0 | 54 | - | - | 12 | - | - |
| Faggiano *et al.* | 25 | 7 | 16 | 4 | 12 | 5 | 3 | 2 | 4 | 0 | 4 |
| Fraisse *et al.* | 92 | 26 | 0 | 0 | 0 | 43 | - | - | 49 | - | - |
| Galeano-Valle *et al.* | 785 | 18 | - | - | - | - | - | - | - | - | - |
| Grandmaison *et al.* | 58 | 9 | - | - | - | - | - | - | - | - | - |
| Grillet *et al.* | 280 | 23 | - | - | - | - | - | - | - | - | - |
| Helms *et al.* | 150 | 28 | 0 | 0 | 0 | 105 | - | - | 45 | - | - |
| Hippensteel *et al.* | 91 | 5 | - | - | - | - | - | - | - | - | - |
| Klok *et al.* | 184 | 66 | 0 | 0 | 0 | 167 | 63 | 104 | 17 | 3 | 14 |
| Llitjos *et al.* | 26 | 6 | 0 | 0 | 0 | 8 | - | - | 18 | - | - |
| Lodigiani *et al.* | 388 | 12 | - | - | - | - | - | - | - | - | - |
| Longchamp *et al.* | 25 | 8 | 1 | 1 | 0 | 22 | 7 | 15 | 2 | 0 | 2 |
| Maatman *et al.* | 109 | 5 | 2 | - | - | 103 | - | - | 4 | - | - |
| Mazzaccaro *et al.* | 32 | 21 | - | - | - | - | - | - | - | - | - |
| Middeldorp *et al.* | 198 | 27 | - | - | - | 167 | 27 | 140 | 19 | 0 | 19 |
| Nahum *et al.* | 34 | 9 | 0 | 0 | 0 | 34 | 9 | 25 | 0 | 0 | 0 |
| Pavoni *et al.* | 40 | 7 | 0 | 0 | 0 | 40 | 7 | 33 | 0 | 0 | 0 |
| Poissy *et al.* | 107 | 22 | - | - | - | - | - | - | - | - | - |
| Ren *et al.* | 48 | 5 | 1 | - | - | 47 | - | - | 0 | 0 | 0 |
| Tavazzi *et al.* | 54 | 2 | 0 | 0 | 0 | 54 | 2 | 52 | 0 | 0 | 0 |
| Thomas *et al.* | 62 | 5 | 0 | 0 | 0 | 62 | 5 | 57 | 0 | 0 | 0 |
| Voicu *et al.* | 56 | 13 | 0 | 0 | 0 | 49 | - | - | 7 | - | - |
| Zhang *et al.* | 143 | 23 | 90 | - | - | 53 | - | - | 0 | 0 | 0 |
| Zotzmann *et al.* | 10 | 3 | 0 | 0 | 0 | 10 | 3 | 7 | 0 | 0 | 0 |

VTE = venous thromboembolism.

- = not available.

# Additional figure 1. Funnel plot of:

## 1a studies in medical ward ± ICU inpatients;

Egger test p=0.02

## 1b studies in ICU only.

Egger test p=0.47

# Additional figure 2. Forrest plot of the estimated incidence of proximal DVT:

## 2a stratified by medical ward and ICU;

DVT = deep vein thrombosis, ICU = intensive care unit, TPX = thromboprophylaxis, AC = therapeutic anticoagulation, NA = not available.

## 2b stratified by location and screening.

TPX = thromboprophylaxis, AC = therapeutic anticoagulation, NA = not available, ICU = intensive care unit.

# Additional figure 3. Forrest plot of the estimated incidence of PE:

## 3a stratified by general ward and ICU;

PE = pulmonary embolism, ICU = intensive care unit, TPX = thromboprophylaxis, AC = therapeutic anticoagulation, NA = not available.

## 3b stratified by location and screening.

TPX = thromboprophylaxis, AC = therapeutic anticoagulation, NA = not available, ICU = intensive care unit.

# Additional figure 4. Forrest plot of the meta-analytic risk of VTE, restricted to medical inpatients without ICU stay.

VTE = venous thromboembolism, ICU = intensive care unit, TPX = thromboprophylaxis, AC = therapeutic anticoagulation, DVT = deep vein thrombosis, PE = pulmonary embolism, NA = not available.

# Additional figure 5. Sensitivity analysis, restricting to high-quality studies:

## 5a stratified by location ;

## 5b stratified by location and screening.

TPX = thromboprophylaxis, AC = therapeutic anticoagulation, PE = pulmonary embolism, DVT = deep vein thrombosis, NA = not available, ICU = intensive care unit.
